# Local and Systemic Cytokine Profiling for Pancreatic Ductal Adenocarcinoma to Study Cancer Cachexia in an Era of Precision Medicine

**DOI:** 10.3390/ijms19123836

**Published:** 2018-12-01

**Authors:** Michael H. Gerber, Patrick W. Underwood, Sarah M. Judge, Daniel Delitto, Andrea E. Delitto, Rachel L. Nosacka, Bayli B. DiVita, Ryan M. Thomas, Jennifer B. Permuth, Steven J. Hughes, Shannon M. Wallet, Andrew R. Judge, Jose G. Trevino

**Affiliations:** 1 Department of Surgery, College of Medicine, University of Florida Health Science Center, Gainesville, FL 32610, USA; michael.gerber@surgery.ufl.edu (M.H.G.); patrick.underwood@surgery.ufl.edu (P.W.U.); daniel.delitto@surgery.ufl.edu (D.D.); ryan.thomas@surgery.ufl.edu (R.M.T.); steven.hughes@surgery.ufl.edu (S.J.H.); 2 Department of Physical Therapy, University of Florida Health Science Center, Gainesville, FL 32610, USA; smsenf@phhp.ufl.edu (S.M.J.); ADelitto@dental.ufl.edu (A.E.D.); rnoscaka@ufl.edu (R.L.N.); arjudge@phhp.ufl.edu (A.R.J.); 3 Department of Neurosurgery, College of Medicine, University of Florida Health Science Center, Gainesville, FL 32610, USA; Bayli.DiVita@neurosurgery.ufl.edu; 4 North Florida/South Georgia Veterans Health System, Department of Surgery, University of Florida College of Medicine, Gainesville, FL 32610, USA; 5 Departments of Cancer Epidemiology and Gastroinestinal Oncology, Moffitt Cancer Center, Tampa, FL 33612, USA; jenny.permuth@moffitt.org; 6 Department of Foundational Sciences, School of Dental Medicine, East Carolina University, Greenville, NC 27834, USA; wallets18@ecu.edu

**Keywords:** pancreatic cancer, cachexia, cytokines, innate immune system

## Abstract

Cancer cachexia is a debilitating condition seen frequently in patients with pancreatic ductal adenocarcinoma (PDAC). The underlying mechanisms driving cancer cachexia are not fully understood but are related, at least in part, to the immune response to the tumor both locally and systemically. We hypothesize that there are unique differences in cytokine levels in the tumor microenvironment and systemic circulation between PDAC tumors and that these varying profiles affect the degree of cancer cachexia observed. Patient demographics, operative factors, oncologic factors, and perioperative data were collected for the two patients in the patient derived xenograft (PDX) model. Human pancreatic cancer PDX were created by implanting fresh surgical pancreatic cancer tissues directly into immunodeficient mice. At PDX end point, mouse tumor, spleen and muscle tissues were collected and weighed, muscle atrophy related gene expression measured, and tumor and splenic soluble proteins were analyzed. PDX models were created from surgically resected patients who presented with different degrees of cachexia. Tumor free body weight and triceps surae weight differed significantly between the PDX models and control (*P* < 0.05). Both PDX groups had increased atrophy related gene expression in muscle compared to control (FoxO1, Socs3, STAT3, Acvr2b, Atrogin-1, MuRF1; *P* < 0.05). Significant differences were noted in splenic soluble protein concentrations in 14 of 15 detected proteins in tumor bearing mice when compared to controls. Eight splenic soluble proteins were significantly different between PDX groups (*P* < 0.05). Tumor soluble proteins were significantly different between the two PDX groups in 15 of 24 detected proteins (*P* < 0.05). PDX models preserve the cachectic heterogeneity found in patients and are associated with unique cytokine profiles in both the spleen and tumor between different PDX. These data support the use of PDX as a strategy to study soluble cachexia protein markers and also further efforts to elucidate which cytokines are most related to cachexia in order to provide potential targets for immunotherapy.

## 1. Introduction

Pancreatic cancer is a lethal malignancy with overall 7% five-year survival. It is projected to become the second leading cause of cancer related deaths by 2030 [1]. Over 80% of patients with pancreatic ductal adenocarcinoma (PDAC) present with some degree of cancer-related muscle wasting or cancer cachexia [2,3]. Cancer-associated cachexia is most prevalent in PDAC and these patients suffer from the greatest weight loss and disability when compared to other cancers [4]. While no universal definition or classification system of cancer cachexia is routinely used in clinical practice, it is characterized by unintentional weight loss due to wasting of muscle and adipose tissue. It is a multifactorial, systemic syndrome associated with progressive functional decline, decreased quality of life, limitations in treatment options, and subsequent worse survival [5,6,7,8]. The degree to which disease burden correlates with cancer cachexia varies widely and suggests that there may be unappreciated underlying cellular and molecular mechanisms contributing to this process [9]. It remains unclear which mechanisms are most important in driving this process. Further, no current therapies exist for this debilitating condition.

Although the mechanisms of cancer cachexia are not yet fully elucidated, there is breakdown of fat by lipolysis, depression of protein synthesis in the muscle, and an increase in muscle catabolism [5,6]. Due to preservation of the non-muscle protein compartment, cancer cachexia is different from the simple starvation of anorexia [10], and it cannot be reversed by nutritional support alone [3,11,12]. Cancer cachexia more likely resembles cachexia seen in patients with infection and injury than starvation [5,13]. The immune response in the tumor microenvironment systemically appears to play a key role. A number of circulating pro-inflammatory cytokines have been implicated in pancreatic cancer cachexia including interleukin 6 (IL-6), interleukin 8 (IL-8), transforming growth factor beta (TGF-β), tumor necrosis factor alpha (TNFα), and monocyte chemoattractant protein-1 (MCP-1) [14,15,16,17]. The source of these cytokines and ultimate systemic effect is unclear and likely multifactorial. Furthermore, differences in cytokine profiles between the tumor microenvironment and systemic circulation and any associations with cachexia in PDAC have not been evaluated. In an era of precision medicine, these cytokine profiles may hold value as a diagnostic and prognostic tool, towards understanding metastatic potential, and preventing cancer cachexia.

In this study, we aimed to use primary patient derived xenograft (PDX) models to better understand cytokine profiles in the tumor microenvironment and systemic circulation and the relationship to cancer cachexia. We hypothesize that there are fundamental differences in cytokine production in the tumor microenvironment and systemic circulation between PDAC tumors. We further hypothesized that differences in these unique profiles will affect the cachectic phenotype.

## 2. Results

### 2.1. Xenograft Selection

Xenografts were created from patients and expanded in vivo using our PDX model described in methods. Patient demographics, operative factors, oncologic factors, and perioperative data for each patient are listed in Table 1. Both patients were Caucasian females with similar weights and body mass index (BMI). Patient G68 had 25 pound weight loss in the 6 months prior to surgery versus 10 pound weight loss in patient G59. The pre-operative computed tomography scan also showed a smaller psoas index in patient G68, a known predictor of survival and consistent with more profound cachexia [8]. Both patients had T3N1 moderately differentiated pancreatic adenocarcinoma in the head of the pancreas and both received a pancreatoduodenectomy for resectable disease. Neither received neoadjuvant chemotherapy. Patient G68, the more cachectic patient, suffered from major morbidity post operatively and succumbed to her disease 169 days post operatively versus 711 days post operatively in the less cachectic patient, G59.

### 2.2. PDX Model Preserves Cancer-Associated Cachexia

**Figure 1:**
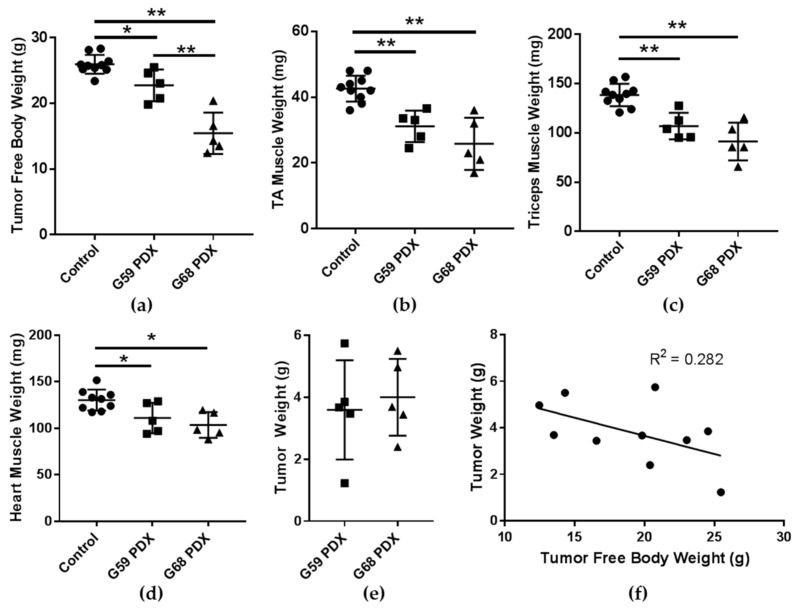
(**a**) Tumor free body weight (TFBW) calculated by (body weight–tumor weight); (**b**) tibialis anterior (TA) muscle weights; (**c**) triceps muscle weights; (**d**) heart muscle weights; (**e**) tumor weights; and (**f**) correlation between tumor free body weight and tumor weight. * *P*-value < 0.05; ** *P*-value < 0.005; TA tibialis anterior.

After successful engraftment of two different patient tumors, we passaged each into the flanks of five mice and compared them to ten age and sex matched control mice. At the study endpoint (84 days), all mice were euthanized and tissue collected. At time of euthanasia, the tumor free body weight (TFBW: calculated as mouse weight – tumor weight) was significantly decreased in both the G59 and G68 PDX groups compared to controls and was significantly less in the G68 groups compared to the G59 group (Figure 1a). The tibialis anterior (TA) muscles, triceps surae muscles, and heart muscle weights at endpoint were decreased in both PDX groups compared to controls (Figure 1b–d). The tumor weight was not different between PDX groups (Figure 1e) and was not significantly correlated with tumor free body weight, *R*^2^ = 0.282 (Figure 1f). Unlike the widely used Lewis lung carcinoma (LLC) and colon-26 (C26) models but similar to patients, the cachectic response is not dependent of the size of the tumor. These data demonstrate a more cachectic phenotype in the G68 PDX group as it had decreased tumor free body weight compared to the G59 PDX group.

### 2.3. Atrophy Related Genes Are Expressed in PDX Models

**Figure 2:**
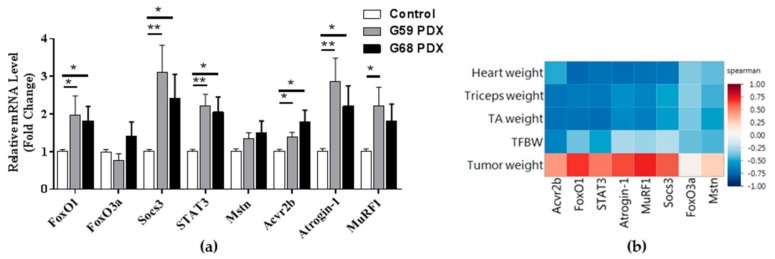
(**a**) Atrophy related gene mRNA expression in tibialis anterior muscle; (**b**) Spearman’s correlations of atrophy related gene expression and mouse patient derived xenograft (PDX) weights; * *P*-value < 0.05; ** *P*-value < 0.005.

The TA muscles were then analyzed for atrophy related gene expression in each mouse. The mRNA levels of Forkhead Box O1 (FoxO1), suppressor of cytokine signaling 3 (Socs3), signal transducer and activator of transcription 3 (STAT3), Activin-R2b, and Atrogin-1 were increased in both PDX groups compared to controls, and MuRF1 was higher in the G59 PDX group compared to controls (Figure 2a). A heat map (Figure 2b) compares the expression of atrophy related genes to the various weights in the PDX cohorts. All eight atrophy genes were negatively associated with PDX muscle weights meaning positively associated with the loss of muscle mass, although these were not significant.

### 2.4. PDX Model Confers Unique Splenic Soluble Protein Profiles

To determine the circulating immune protein profile, we tested mouse splenic concentrations for 38 human soluble cytokines, chemokines, and growth factors. Detectable levels of 15 different soluble factors were found in the splenic lysates. These concentrations were normalized to the total protein of the lysate, and the values can be seen in Table 2. Known factors involved in cachexia such as interleukin 1 alpha (IL-1α), interleukin 1 beta (IL-1β), IL-6, and TNFα were not detected in splenic lysates with this primary PDX model. Of the 15 soluble proteins detected, eight were significantly different between controls and the G59 PDX group while 13 were significantly different between the controls and G68 PDX group. Between the G59 and G68 PDX groups, there were eight significantly different soluble proteins.

Inflammatory factors detected within splenic lysates include interferon alpha-2 (IFNα2), interferon gamma (IFNγ), interleukin 1 receptor antagonist (IL-1RA), interleukin 4 (IL-4), interleukin 7 (IL-7), interleukin 8 (IL-8), interleukin 17a (IL-17A), and interferon gamma-induced protein 10 (IP-10), also known as C-X-C motif chemokine 10 (CXCL10) of which several may play a role in cachexia. Both IFNγ and IL-8 were significantly different between tumor bearing PDX groups. Interestingly, IFNγ is detected at lower levels in the more cachectic G68 PDX group even though this group has significantly higher IP-10 levels. The chemokines detected include fractalkine, growth regulated oncogene (GRO), and macrophage-derived chemokine (MDC). There was variation between production of these proteins between the two PDX groups with both fractalkine and MDC being significantly higher in the less cachectic G59 PDX group. The growth factors detected included fibroblast growth factor 2 (FGF-2), vascular endothelial growth factor (VEGF), FMS-like tyrosine kinase 3 ligand (Flt-3L), and epidermal growth factor (EGF). Growth factors were all significantly decreased in the PDX groups compared to controls.

### 2.5. PDX Model Confers Unique Tumor Microenvironment Soluble Protein Profiles

We then focused on local tumor signaling and analyzed the local tumor soluble immune protein profiles for the same 38 human proteins in tumor lysates of which 21 were detected and are summarized in Table 3. Of the 21 proteins detected in the tumor lysates, 15 were significantly different between the PDX groups with all except granulocyte-macrophage colony-stimulating factor (GM-CSF) being higher in the less cachectic G59 PDX group. IL-1β, a known cachectic factor, was again not detected. IL-1α, IL-6, and TNFα, which were not detected in the splenic lysates, were detected in the tumor lysates.

The pro-inflammatory factors found at significantly higher concentrations in the G59 PDX group include IFNα2, IL-7, and IFNγ, but no difference was found in IL-8 levels. The chemokines found to be different between groups were GRO and MDC. Transforming growth factor alpha (TGFα), FGF-2, and EGF were increased in the G59 PDX group compared to the G68 PDX group suggesting a more angiogenic tumor profile in the less cachectic G59 PDX group. Granulocyte-colony stimulating factor (G-CSF) was significantly increased in the G59 PDX group while GM-CSF was significantly increased in the more cachectic G68 PDX group.

IL-1α and IL-1β are known factors associated with cachexia and while there are no significant differences between these molecules in our PDX model, the less cachectic G59 PDX group had significantly higher concentrations of IL-1RA which blocks both IL-1 receptors thus inhibiting the downstream effects of both IL-1α and IL-1β.

### 2.6. Splenic and Tumor Soluble Protein Associations

**Figure 3:**
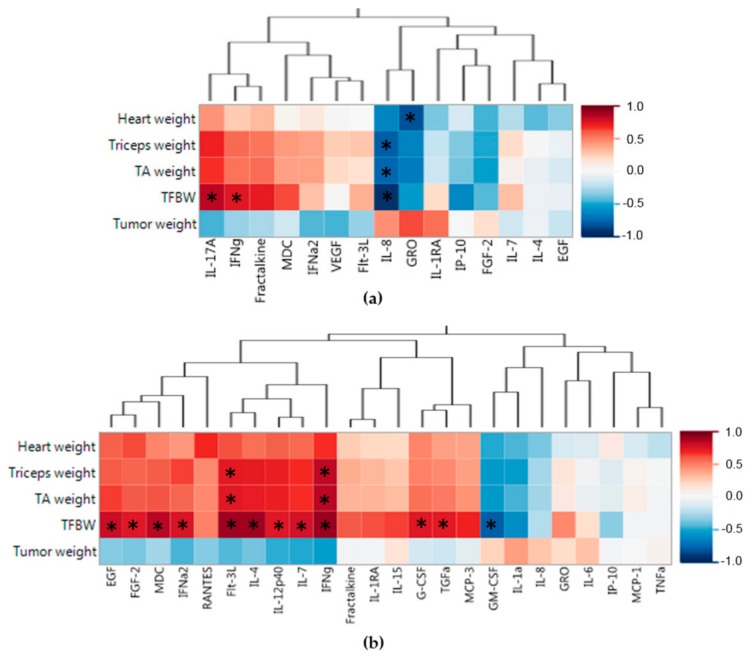
PDX splenic and tumor soluble protein associations with mouse PDX weights. (**a**) Spearman’s correlations of PDX weights with splenic soluble protein profiles. (**b**) Spearman’s correlations of PDX weights with tumor soluble protein profiles. Red indicates a positive association and blue indicates a negative association. Proteins are clustered based on Spearman’s correlations. * *P*-value < 0.01; TA (tibialis anterior), TFBW (tumor free body weight).

We next sought to find associations with the soluble protein profiles and the associated PDX muscle weights, TFBW, and tumor weight. The heat map in Figure 3a shows associations of the splenic soluble proteins with the PDX weights using all 10 tumor bearing mice from both G59 and G68 PDX groups. Figure 3a exposes distinct sets of associations with the left side of the heat map containing proteins positively associated with muscle and body weight and negatively associated with tumor weight, of which two were significant (IL-17A, IFNγ; *P* < 0.01), suggesting these proteins may be associated with a protective effect from cancer cachexia. Proteins that have a negative association with muscle and body weight are clustered on the right side of the heatmap with IL-8 having significant associations with tumor free body weight and skeletal muscle weight (*P* < 0.01) and GRO having significant association with heart muscle weight. These two proteins may be involved in the systemic effect of the immune system that promotes muscle wasting. Again, it is important to note that no protein had a significant association with tumor weight.

The heat map in Figure 3b demonstrates associations between the tumor soluble proteins and the PDX weights. Distinct protein groups can be seen in the heat map, with the left sided protein cluster having a positive association with muscle and body weights, of which two proteins had more than one significant association (Flt-3L, IFNγ; *P* < 0.01). This suggests that production of these proteins in the tumor may create downstream indirect effects that protects from muscle wasting. The right sided cluster of proteins in the heat map shows local tumor factors that are associated with muscle and weight loss of which only GM-CSF (*P* < 0.01) was significantly correlated with weight loss, possibly creating some local tumor effect that causes systemic weight loss.

**Figure 4:**
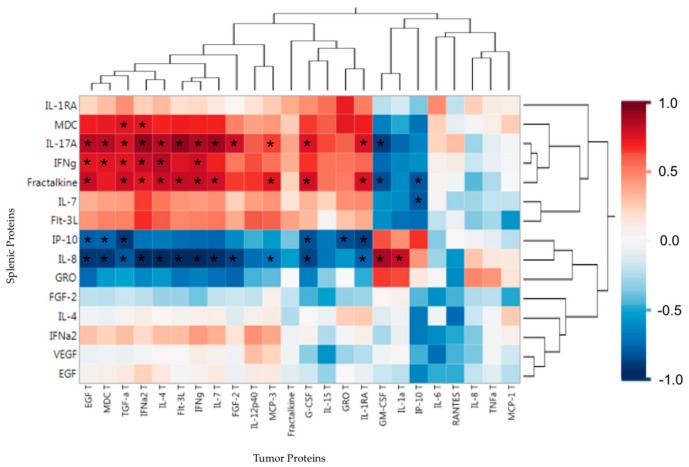
PDX splenic soluble protein associations (Spearman’s correlation coefficients) with tumor soluble proteins. Red indicates a positive association and blue indicates a negative association. Proteins are clustered based on Spearman’s correlations. * *P*-value < 0.01.

After making the comparison of splenic and tumor proteins to the PDX mice weights, we analyzed the correlations between the splenic protein and tumor protein profiles to look for possible tumor proteins that have an effect on the systemic soluble protein profile. In the Figure 4 heat map, we see two splenic proteins (IL-17A, IFNγ) which had positive correlations with TFBW, also show a strong positive correlation with four of the five tumor proteins that had the strongest associations with increased TFBW (IL-4, Flt-3L, IFNγ, IL-7) and skeletal muscle weights (Flt-3L, IFNγ) in this study. Splenic proteins that were associated with increased TFBW are all negatively correlated with tumor GM-CSF, a tumor protein associated with weight loss in the PDX mice. Splenic IL-8 was associated with weight and muscle loss and had a negative association with the same tumor proteins associated with increased TFBW (IL-4, Flt-3L, IFNγ, IL-7) and skeletal muscle weights (Flt-3L, IFNγ). Splenic IL-8 also has a strong positive correlation to tumor GM-CSF and IL-1α. Tumor GM-CSF was associated with increased cachexia in the PDX mice.

Interestingly, splenic IP-10 had the opposite correlation profile of splenic IFNγ. As IP-10 should be induced by IFNγ [18], we expected these two proteins to have similar correlations with other proteins, however, the opposite was observed. These data suggest that systemic IL-17A and IFNγ may protect from weight loss in cancer cachexia and may be induced by a particular tumor microenvironment protein profile that includes Flt-3L, IL-4, IFNγ, IL-7. These data also suggest that systemic IL-8 may induce cancer cachexia by increasing muscle wasting and weight loss and could be induced by a tumor protein profile that has high levels of GM-CSF.

**Figure 5:**
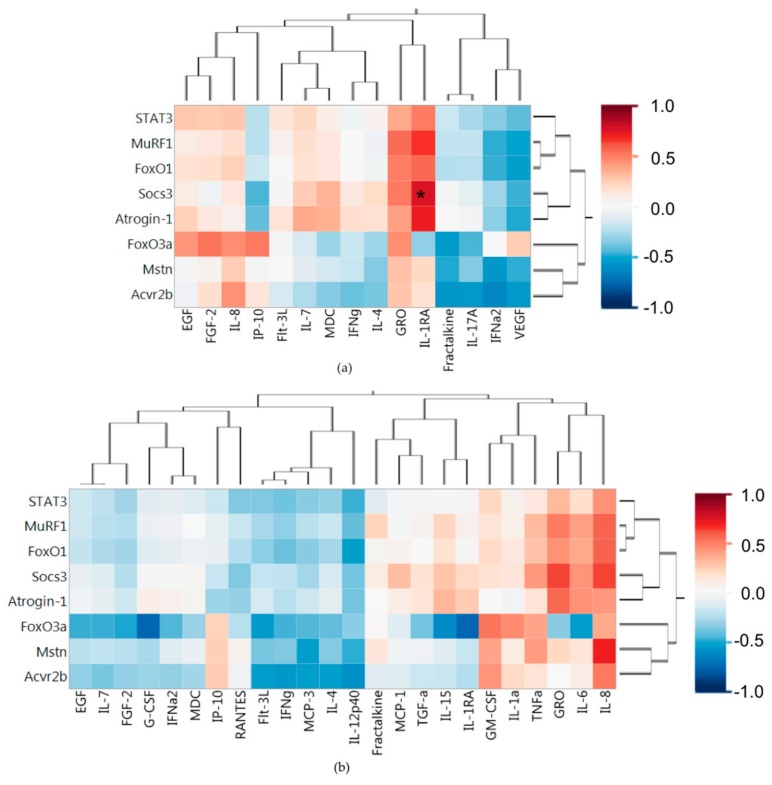
PDX mRNA associations (Spearman’s correlation coefficients) with (**a**) splenic and (**b**) tumor soluble proteins. Proteins and genes are clustered based on Spearman’s correlations. Red indicates a positive association and blue indicates a negative association. * *P*-value < 0.01.

The immune protein profiles were compared to the atrophy associated gene expression in muscle. Figure 5a shows associations between splenic proteins and the TA muscle atrophy related gene expression. The only splenic protein with a significant association, positive or negative, with atrophy related gene expression was IL-1RA which was positively associated with Soc3. When looking at the splenic proteins that were associated with increased TFBW (IFNγ, IL-17A), we see no significant associations with the atrophy genes. Splenic IL-8, which was associated with muscle loss and weight loss, also had a positive association with all the atrophy related genes, but no significant associations. Figure 5b shows associations of the tumor proteins with atrophy gene expression in muscle. No significant correlations were found in the tumor proteins compared to the atrophy related gene expression in the TA muscles. The two tumor proteins (Flt-3L, IFNγ) positively associated with muscle weight and TFBW both had negative correlations with all atrophy related gene expression. Tumor GM-CSF and IL-1α which were negatively associated with muscle weight and TFBW both had weak positive association with atrophy related gene expression. Tumor GRO and IL-8, which did not have strong correlations with weights, had stronger correlations with atrophy related gene expression.

## 3. Discussion

Patient derived xenografts exhibited significant reductions in tumor free body weight and muscle weight compared to control mice, consistent with a cachectic phenotype. The tumor from the more cachectic patient appeared to recapitulate more severe cachexia in the PDX model. The objective of this work was to better understand cytokine profiles in the tumor microenvironment and systemic circulation and how they related to cachexia in a representative model of human pancreatic adenocarcinoma. There were significant differences in cytokine profiles of splenic lysates between control and PDX groups. Furthermore, there were distinct cytokine profiles in the tumor microenvironment of the two PDX groups highlighting the heterogeneity of individual pancreatic cancer patients. The profiles differed at the local and systemic level. As hypothesized, certain local and systemic cytokines were associated with TFBW and muscle weights. Furthermore, the tumor cytokines associated with weight loss were different from the splenic cytokines associated with weight loss. A number of cytokines were associated with a protective effect on muscle and body weight. Splenic proteins associated with muscle and body weight preservation in the pancreatic cancer PDX mice (IFNγ, IL-17A) were produced at higher levels in the less cachectic PDX group and splenic IL-8, associated with body weight loss, was produced at higher levels in the more cachectic PDX group. Taken together, this data suggests that local and systemic cytokine profiles play unique roles in the development of cachexia, some of which appear protective and others more pathogenic.

Closer evaluation of the systemic and tumor microenvironment cytokine profiles demonstrates that these cytokines may be playing a key role in the development of cachexia. IL-8 has long been thought to be involved in cachexia [5,6,19,20,21]. This pro-inflammatory cytokine can be produced from the tumor inciting an acute phase response from the liver or it can be induced in hepatocytes that can then increase the acute phase response of the liver promoting cachexia [19]. Splenic lysates demonstrated significantly elevated IL-8 levels in the PDX mice compared to controls. The more cachectic PDX group, G68, had elevated IL-8 levels in tumor tissue compared to G59. Fractalkine, GRO, IFNα2, IFNγ, IL-4, and MDC are also thought to play a role in the development of cancer cachexia. Fractalkine protects myotubes from TNFα induced damage in muscle and may protect against cancer cachexia [22,23]. Fractalkine was significantly lower in the more cachectic patient PDX group. IL-4 was lower in the more cachectic G68 group. IL-4 mRNA has previously been shown to be downregulated in the liver of pancreatic cancer patients suffering from cachexia [24]. GRO is known to increase muscle fatty acid oxidation contributing to the breakdown of muscle [25]. This molecule is also known to be produced by the liver in response to increased levels of IL-6 [26]. We see an increase in systemic GRO from controls in both G59 and G68 PDX groups. The elevation in systemic GRO could be at least partially responsible for systemic loss of fat and loss of muscle mass. Type I interferons increase fatty acid oxidation and oxidative phosphorylation as well as increase Socs3 and Atrogin-1 gene expression [27,28]. However, in a model of renal cell carcinoma, it was shown to prevent weight loss [29]. The more cachectic PDX group showed a significant decrease in systemic IFNα2 compared to controls. There was a significant decrease in FGF-2 and VEGF in both PDX groups, indicative of downregulation of systemic angiogenesis.

There were associations between atrophy related gene expression and TFWB and muscle weight. FoxO1, which was significantly elevated in our PDX models, is a transcription factor which increases the transcription of atrophy-related genes associated with both the ubiquitin proteasome pathway, including atrogin-1 and MuRF1, and the autophagy/lysosomal pathway. FoxO1 is also known to regulate gluconeogenesis and glycogenolysis by insulin signaling as well as mediates pro-inflammatory cytokines in response to high glucose, TNFα, and lipopolysaccharide stimulation [30]. Atrogin-1 and MuRF1 are both muscle-specific E3 ubiquitin ligases, a family of proteins important to the degradation of proteins and which play a key role in cancer cachexia [31]. Atrogin-1 was elevated in both PDX groups and MuRF-1 was elevated above control in the G59 PDX group. The gene expression of the transcription factor STAT3 and Socs3 which is a STAT induced STAT inhibitor were both elevated in the PDX groups indicating that the muscle is a target of an active inflammatory signal [32]. Myostatin, a member of the TGFβ superfamily and myocyte produced myokine, and its receptor Acvr2b which binds to several members of the TGFβ superfamily, both inhibit myogenesis [33]. Myostatin expression was not significantly elevated in either PDX groups, but its receptor Acvr2b was increased in both PDX groups indicating there may be TGFβ signaling acting on the muscle. All the atrophy related genes had negative associations with TFBW and TA, triceps, and heart muscle weight. In particular, Acvr2b, FoxO1, and STAT3 all had spearman correlations of at least 0.63 with three of these weights indicating that both the transcriptions factors and TGFβ signaling may play a role in pancreatic cancer associated cachexia.

NSG mice lack an adaptive immune system as well as natural killer cells. The immune system signaling is thought to be the main driver behind cachexia. As this model lacks a large component of the immune system, one might not expect to see a cachectic syndrome in these tumor bearing mice. However, this PDX model shows that the tumor-bearing mice do indeed develop cancer cachexia, suggesting the adaptive immune system is not necessary for cancer cachexia to develop. The role of the adaptive immune system remains uncertain. Known factors involved in cachexia, IL-1α, IL-1β, IL-6, IL-8 and TNFα are all proteins that may be produced by the innate immune cells or other non-immune cell types such as epithelial cells or stromal cells [5,34]. The innate immune system may be the primary driver behind cancer cachexia. Of these proteins, our study found detectable levels of IL-8, indicating this may be one of the main drivers in pancreatic cancer induced cachexia. Other groups have shown that IL-8 is associated with increased cachexia in gastric, prostate, esophageal, and pancreatic cancer patients [21,35,36,37].

Our study has several limitations. First, we used two different patient tumors to generate our PDX model. As suggested by our results, each patient tumor has a unique cytokine profile. These data may not be representative of all patients and thus emphasizes the importance of individualized, precision medicine. The PDX were implanted in the flanks of mice. It is possible that orthotopically implanted PDX would have yielded different results. The atrophy related gene expression was limited to the genes we tested. There could be other, more important genes playing a key role in muscle atrophy. Adipose wasting, an important component of cancer cachexia, was not directly measured but may play a role in the observed difference in weights. This is supported by the differences in body weights between PDX models but similar muscle weights. The cytokines tested remain limited to the 38 cytokines available in our assay. There are numerous other cytokines which could play a key role in cancer cachexia. Further, there is often cross-reactivity between human and murine cytokines detected by this kit. Despite this limitation, we have shown that cytokine profiles differ between tumors and that this likely plays a role in the development of cancer cachexia. As noted, above, the NSG mice lack an adaptive immune system and have deficient innate immunity in that they have defects in macrophage and natural killer cells. It is unclear what role the adaptive immune system may play in development of cancer cachexia. A syngeneic mouse model would necessitate use of murine PC cell lines or murine derived tumor. This model would lack genetic heterogeneity and be limited to murine cytokines. The PDX model offers the ability to assess human pancreatic cancer tissue and human cytokines. In an era of increasing interest in personalized medicine, it allows for implantation of PDX from multiple patients to provide genetic diversity.

In conclusion, our PDX model recapitulates the varying cachectic phenotypes between pancreatic cancer tumors. In our study, the degree of cachexia is unrelated to tumor burden. Rather, it may be related to immune interplay with the tumor at the local and systemic level. There are unique systemic and local cytokine profiles between the different PDX and control groups. It is likely that some cytokines only act locally and cause upregulation of other cytokines which have a systemic effect. Specific cytokines at the local and systemic levels are associated with weight loss in this study. These cytokines may be driving expression of atrophy related genes in the muscle, leading to cachexia. There are also likely further systemic actions of these cytokines, which were not measured, and warrant further investigation. Future work to evaluate for differences in serum cytokine profiles could improve the clinical applicability of cytokine profiling. Further work to evaluate cytokine profiles offers a novel concept for studying cancer cachexia. If particular local and systemic cytokine profiles, rather than individual cytokines, can be identified as more protective or pathologic towards cachexia, it will allow for better understanding of the pathways involved and targeting with immunotherapies.

## 4. Materials and Methods

### 4.1. Patient Derived Xenograft Model

The study was approved by the University of Florida Institutional Review Board (IRB201600873) and animal studies were performed with approval from the University of Florida Institutional Animal Care and Use Committee (IACUC 201706590, 30 November 2017). Human tumor tissue specimens were collected at the time of operation from excess tumor tissue, cut into 3-mm pieces and implanted into the flanks of NOD.Cg-Prkdc^scid^ IL2rg^tm1Wjl^/SzJ (NSG) mice from Jackson Laboratory (Bar Harbor, ME, USA). Xenografts were allowed to grow to a diameter of 2.0 cm before passage into other age- and sex-matched NSG mice. Each group of mice was simultaneously euthanized once IACUC mandated tumor endpoint was reached.

### 4.2. Mouse Tissue Harvest and Processing

At the time of euthanasia, tumor, spleen, TA, and triceps surae complexes (gastrocnemius, soleus, and plantaris) were harvested for weights, histology, and molecular analysis. Tissues were rinsed in phosphate buffered saline (PBS), weighed, and snap frozen in liquid nitrogen or embedded in Optimal Cutting Temperature (OCT) medium.

### 4.3. Genetic Expression Profiling

Mouse PDX TA muscles were homogenized in Trizol and RNA isolated. cDNA was synthesized from equal amounts of RNA using Ambion’s RETROscript FirstStrand Synthesis Kit (Life Technologies, Grand Island, NY, USA). qPCR was performed using 7300 real-time PCR system and TaqMan Gene Expression Assays (Applied Biosystems, Austin, TX, USA) for forkhead box O1 (FoxO1, NM_019739.3), FoxO3a (NM_019740.2), Suppressor of cytokine signaling (Socs3, NM_007707.3), signal transducer and activator of transcription 3 (STAT3, NM_011486.4), myostatin (Mstn, NM_010834.2), activin receptor 2b (Acvr2b, NM_007397.2), atrogin-1 (NM_026346.3), and muscle RING-finger protein 1 (MuRF-1, NM_001039048.2).

### 4.4. Soluble Protein Analysis

Tumor and splenic tissues were dissociated mechanically and homogenized in cell lysis buffer (Cell Signaling #9803, Danvers, MA, USA) containing protease inhibitors (Cell Signaling #5872, Danvers, MA, USA). Homogenates were centrifuged to separate insoluble debris from the protein containing supernatant. Each supernatant protein concentration was determined using a protein quantification kit (Bio-Rad, Hercules, CA, USA). The supernatants were analyzed by Milliplex Premixed 38-Plex Immunology Multiplex Assays (Millipore, Merk KGaA, Darmstadt, Germany) according to the manufacturer’s protocol and analyzed with Milliplex software. Cytokine concentrations were normalized to total protein concentrations of the supernatant. The final values are expressed as (pg of cytokine)/(mg of total protein).

### 4.5. Statistical Analysis

Statistical analyses were performed in JMP Pro 12 (Cary, NC, USA). To assess for differences in two quantitative variables, T tests were performed. In the case of three quantitative variables, one way ANOVA with multiple comparisons was performed. Spearman correlations were performed to assess for associations between atrophy related genes, splenic soluble proteins, and tumor soluble proteins. When applicable, results were graphed using GraphPad Prism version 7 (San Diego, CA, USA).

## Figures and Tables

**Table 1:** Patient demographics and operative/oncologic outcomes.

Patient Demographics	G59	G68
Age	73	64
Charlson comorbidity index	5	4
Race	White	White
Sex	Female	Female
BMI	27.3	29.2
Weight at time of surgery (pounds)	149	170
Weight loss prior to surgery (pounds)	10 (6.3%)	25 (12.8%)
Psoas Index at time of surgery	0.639	0.526
Neoadjuvant therapy	No	No
**Operative & Oncologic Factors**		
Pathologic diagnosis	Adenocarcinoma	Adenocarcinoma
EBL	600	200
Transfusion, intra-op	No	No
Transfusion, post-op	No	Yes
Portal vein resection	No	No
Total lymph nodes	15	18
Positive lymph nodes	4	2
Tumor size (cm)	Indeterminate	3.8
Margins	Uninvolved	Uninvolved
Differentiation	Moderate	Moderate
Stage	T3N1	T3N1
**Postoperative Complications**		
Length of stay (days)	7	74
Pancreatic leak	No	Yes
Post pancreatectomy hemorrhage	No	Yes
Major morbidity (clavien III/IV)	No	Yes
Death (Postoperative day)	711	169

**Table 2:** Detectable splenic lysate proteins.

Splenic Lysate Proteins	Control (*n* = 9)	G59 (*n* = 5)	G68 (*n* = 5)	Control vs. G59	Control vs. G68	G59 vs. G68
	Mean (SD)	Mean (SD)	Mean (SD)	*P*-Value	*P*-Value	*P*-Value
FGF-2	2587.5 (557.5)	1491.0 (398.6)	1690.2 (552.9)	**0.002**	**0.013**	0.532
VEGF	75.1 (12.0)	9.7 (7.7)	8.9 (3.8)	**<0.001**	**<0.001**	0.846
Fractalkine	56.6 (28.8)	67.8 (24.3)	19.8 (13.7)	0.478	**0.021**	**0.005**
IL-8	1.5 (1.1)	17.4 (15.2)	155.5 (113.9)	**0.007**	**0.001**	**0.028**
GRO	1.4 (1.2)	108.2 (51.0)	158.6 (66.1)	**<0.001**	**<0.001**	0.214
MDC	21.7 (14.9)	25.7 (7.9)	3.0 (1.4)	0.590	**0.017**	**<0.001**
IFNα2	15.3 (3.7)	11.8 (2.5)	10.7 (2.6)	0.080	**0.028**	0.511
IL-7	9.8 (1.9)	8.4 (1.8)	5.7 (1.6)	0.229	**0.002**	**0.031**
Flt-3L	6.4 (3.5)	3.5 (1.1)	2.0 (0.7)	0.107	**0.019**	**0.034**
EGF	4.6 (1.5)	2.8 (1.3)	2.5 (0.9)	**0.040**	**0.013**	0.667
IFNγ	4.3 (2.2)	4.8 (1.6)	0.8 (0.3)	0.663	**0.004**	**0.001**
IP-10	2.7 (1.7)	1.2 (0.9)	4.4 (3.0)	0.090	0.183	**0.049**
IL-17A	2.4 (0.8)	2.0 (0.6)	0.2 (0.3)	0.308	**<0.001**	**<0.001**
IL-4	1.1 (1.1)	2.7 (1.1)	2.3 (0.7)	**0.024**	0.053	0.498
IL-1RA	0.1 (0.3)	20.4 (26.3)	1.4 (0.6)	**0.033**	**<0.001**	0.144

Concentrations in units of pg/mg protein; SD: standard deviation. Data in bold represents *P*-value < 0.05.

**Table 3:** Detectable tumor lysate proteins.

	G59 (*n* = 5)	G68 (*n* = 5)	
	Mean (SD)	Mean (SD)	*P*-value
IL-1RA	1056.9 (198.8)	248.9 (126.9)	**<0.001**
GRO	689.7 (215.8)	293.5 (185.9)	**0.014**
FGF-2	635.8 (359.7)	236.2 (135.2)	**0.049**
IL-8	194.3 (122.0)	267.8 (153.8)	0.426
Fractalkine	121.8 (60.7)	62.9 (12.7)	0.066
RANTES	88.6 (46.4)	82.7 (75.6)	0.885
IP-10	13.1 (6.1)	65.0 (53.0)	0.061
GM-CSF	3.6 (1.5)	16.3 (7.5)	**0.006**
TGFα	15.2 (5.2)	3.6 (2.1)	**0.002**
IL-1α	4.1 (2.9)	11.6 (8.1)	0.085
MDC	7.0 (2.8)	2.4 (1.0)	**0.009**
TNFα	2.9 (1.3)	6.0 (7.2)	0.364
IFNα2	6.6 (1.3)	2.2 (1.4)	**0.001**
IL-15	4.9 (0.6)	3.8 (0.5)	**0.008**
MCP-1	3.4 (3.9)	4.8 (6.5)	0.679
IL-7	3.5 (1.5)	1.5 (0.6)	**0.026**
IL-6	2.9 (2.2)	1.3 (1.0)	0.167
G-CSF	2.8 (0.6)	0.8 (0.4)	**<0.001**
Flt-3L	2.4 (0.5)	0.9 (0.3)	**<0.001**
IFNγ	2.0 (1.0)	0.7 (0.4)	**0.022**
IL-4	1.9 (1.1)	0.5 (0.1)	**0.020**
EGF	1.6 (1.0)	0.5 (0.2)	**0.033**
MCP-3	1.3 (0.9)	0.5 (0.2)	0.083
IL-12p40	1.2 (0.4)	0.6 (0.4)	**0.049**

VEGF above detection limits; Concentrations in units of pg/mg protein; SD: standard deviation. Data in bold represents P-value < 0.05.

## Abbreviations

PDX	Patient Derived Xenograft
PDAC	Pancreatic Ductal Adenocarcinoma
NSG	NOD.Cg-Prkdc^scid^ IL2rg^tm1Wjl^/SzJ
IACUC	Institutional Animal Care and Use Committee
TA	Tibialis Anterior
